# Antioxidant responses under salinity and drought in three closely related wild monocots with different ecological optima

**DOI:** 10.1093/aobpla/plx009

**Published:** 2017-02-21

**Authors:** Mohamad Al Hassan, Juliana Chaura, María P. Donat-Torres, Monica Boscaiu, Oscar Vicente

**Affiliations:** 1 Instituto de Biología Molecular y Celular de Plantas (UPV-CSIC), Universitat Politècnica de València, 46022 Valencia, Spain; 2 Instituto de Investigación para la Gestión Integral de Zonas Costeras (UPV), Universitat Politècnica de València, 46730 Grao de Gandía, Spain; 3 Instituto Agroforestal Mediterráneo (UPV), Universitat Politècnica de València, 46022 Valencia, Spain; 4 Present address: The New Zealand Institute for Plant & Food Research Ltd, Auckland, New Zealand; 5 Permanent address: Department of Biological Sciences, Faculty of Natural Sciences, Universidad ICESI, Cali, Colombia

**Keywords:** Antioxidant enzymes, antioxidant phenolics, ecological adaptation, *Juncus*, malondialdehyde (MDA), photosynthetic pigments, salt stress, water deficiency stress

## Abstract

Some deleterious effects of drought, soil salinity and other abiotic stresses are mediated by the generation of oxidative stress through an increase in reactive oxygen species (ROS) that damage cellular membranes, proteins and DNA. In response to increased ROS, plants activate an array of enzymatic and non-enzymatic antioxidant defences. We have correlated the activation of these responses with the contrasting tolerance to salinity and drought of three species of the genus *Juncus*, viz. *J. maritimus*, *J. acutus* (both halophytes) and *J. articulatus* (salt-sensitive). Both stresses were given for 8 weeks to 6-week-old seedlings in a controlled environment chamber. Each stress inhibited growth and degraded photosynthetic pigments in the three species with the most pronounced effects being in *J. articulatus*. Salt and water stress also generated oxidative stress in all three taxa with *J. articulatus* being the most affected in terms of accumulation of malondialdehyde (a reliable oxidative stress marker). The apparent lower oxidative stress in halophytic *J. maritimus* and *J. acutus* compared with salt-sensitive *J. articulatus* is explained by a more efficient activation of antioxidant systems since salt or water deficiency induced a stronger accumulation of antioxidant phenolic compounds and flavonoids in *J. maritimus* and *J. acutus* than in *J. articulatus*. Qualitative and quantitative differences in antioxidant enzymes were also detected when comparing the three species and the two stress treatments. Accordingly, glutathione reductase and superoxide dismutase activities increased in the two halophytes under both stresses, but only in response to drought in *J. articulatus*. In contrast, ascorbate peroxidase activity varied between and within species according to treatment. These results show the relative importance of different antioxidant responses for stress tolerance in species with distinct ecological requirements. The salt-sensitive *J. articulatus*, contrary to the tolerant taxa, did not activate enzymatic antioxidant responses to salinity-induced oxidative stress.

## Teaser

We studied the level of oxidative stress and the activation of antioxidant responses in three rush species - sea rush (Juncus maritimus), spiny rush (J. acutus) and jointleaf rush (J. articulatus) - subjected to salt and water stress treatments. The halophytes J. maritimus and J. acutus were the most tolerant taxa; they were less affected by oxidative stress than the salt-sensitive J. articulatus under both conditions, due to more efficient activation of enzymatic and non-enzymatic antioxidant systems. Our results show the relative importance of different antioxidant responses for stress tolerance in species with distinct ecological requirements.

## Introduction

Drought and soil salinity are important environmental stress factors that cause large reductions in global agricultural production and greatly influence the distribution of wild species in nature (Dunson and Travis 1991; Bartels and Sunkar 2005). Most plant species cannot tolerate extended drought or saline concentrations over 200 mM NaCl (Hasegawa *et al.* 2000; Flowers and Colmer 2008) because these conditions provoke a series of deleterious effects in the plants that include disturbed cellular osmotic balance, inhibited photosynthesis, inhibition of enzyme activities and cellular processes, and interference with mineral nutrition. These effects lead to slower growth and eventually to plant death (Munns and Termaat 1986; Zhu 2001; Munns and Tester 2008). In addition, these and other abiotic stresses cause oxidative stress through the increased formation of reactive oxygen species (ROS) (Foyer and Noctor 2003; Bose *et al.* 2014; De Carvalho 2013).

ROS typically arise by the transfer to O_2_ of one, two or three electrons, to form superoxide (O_2_^-^), hydrogen peroxide (H_2_O_2_) or the hydroxyl radical (HO^-^), respectively (Rodrigo-Moreno *et al.* 2013). When in excess, each of these is highly cytotoxic, due to their reactivity with various key cellular components (Breusegem *et al.* 2001; Quiles and López 2004; Sharma *et al.* 2012). Small amounts of ROS are by-products of normal cell metabolism, formed in vital processes such as photorespiration, photosynthesis and respiration (Martinez *et al.* 2001; Mittler 2002; Uchida *et al.* 2002). Abiotic stress increases their production resulting in a dramatic promotion of oxidative damage (Asada 2006; Bose *et al.* 2014). High ROS concentrations cause a major disturbance to intracellular ionic homeostasis by depressing cytosolic K^+^ concentrations followed by activation of proteases and endonucleases (Shabala 2009; Demidchik *et al.* 2010), the oxidation of unsaturated fatty acids in lipids (affecting cell membrane integrity), of amino acid residues in proteins (inhibiting enzyme activities and the function of the photosynthetic apparatus) and of DNA. The collective effect can lead to cell death (Yu *et al.* 2011; Kumari *et al.* 2015). Paradoxically, under non-stressful conditions, ROS at low cellular concentrations play an important role as signalling molecules involved in plant growth, development, gravitropism, hormonal action and many other normal physiological processes (Mittler 2002; Apel and Hirt 2004; Mittler *et al.* 2004; Foyer and Noctor 2005; Miller *et al.* 2008, Bose *et al.* 2014). Such low-level ROS functions include triggering of antioxidant defence mechanisms for adapting to abiotic stress (Miller *et al.* 2008; Abogadallah 2010; Jaspers and Kangasjärvi 2010; Kumari *et al.* 2015). For example, several ROS, at concentrations much lower than those causing cellular damage, can activate different Na^+^- and K^+^-permeable ion channels (Demidchik and Maathuis 2007; Demidchik *et al.* 2007; Richards *et al.* 2014) that help maintain the cytosolic K^+^/Na^+^ ratios needed for salinity tolerance (Maathuis and Amtmann 1999; Anschütz *et al.* 2014).

To help avoid excessive ROS accumulation during stress whilst maintaining appropriately small amounts for signalling, plants activate enzymatic and non-enzymatic antioxidant systems. The latter include antioxidant compounds such as ascorbic acid, glutathione, β-carotenes, flavonoids or other phenolic compounds. The commonest enzymatic antioxidant systems are superoxide dismutase (SOD), catalase (CAT), ascorbate peroxidase (APX) (and other peroxidases) or redox regulatory enzymes such as glutathione reductase (GR), among many others (Ozgur *et al.* 2013). Under unfavourable conditions, the biosynthesis of these antioxidant molecules and the activity of these enzymes are altered (Rossel *et al.* 2002; Horling *et al.* 2003; Mittler *et al.* 2004).

Paradoxically, most studies on stress tolerance in plants have been conducted in model species that are not stress tolerant, especially in *Arabidopsis thaliana* and, to a much lesser extent, in crop species (Sanders 2000; Zhu 2001). However, the degree of tolerance to salt or water shortage is not comparable to that attained by wild species adapted, in nature, to particular stressful environments. Although stress tolerance mechanisms are ubiquitous in plants, the molecular and biochemical pathways leading to improved tolerance may act additively or synergistically in plants naturally adapted to arid and/or saline habitats (Sekmen *et al.* 2007; Kumari *et al.* 2015; Srivastava *et al.* 2015).

Therefore, the mechanisms of response operating in stress tolerant taxa can be expected to be more effective than those of non-tolerant species. Consequently, comparative studies of genetically related, stress tolerant and stress sensitive naturally occurring species are gaining increasing attention because of their potential for understanding stress tolerance mechanisms. Salt and drought tolerant species of *Thellungiella* (taxonomically related to *Arabidopsis* since both genera belong to the Brassicaceae) have been proposed as extremophile models for abiotic stress tolerance studies (Inan *et al.* 2004; Gong *et al.* 2005; Kant *et al.* 2006). The complete genome data on *Thellungiella parvula* and *T. salsuginea* have contributed to elucidating a ‘full picture’ of stress responses in dicotyledonous halophytes. Nevertheless, the involvement of ROS and antioxidants in stress tolerance mechanisms of monocot halophytes is still poorly understood (Ozgur *et al.* 2015). Further studies on stress tolerant and stress sensitive, taxonomically related monocot species drawn from natural plant communities exposed to extreme environments are thus of great interest.

The monocotyledonous genus *Juncus* (family Juncaceae) includes a wide range of halophytes and glycophytes and was thus a suitable choice for the present study. Most previous comparative analyses of stress responses in genetically related taxa have been performed either on different crop cultivars, or in dicotyledonous genera.

Three species of the genus *Juncus* with different degrees of tolerance to drought and salt stress were selected. They included two halophytes, *Juncus maritimus* and *J. acutus*, which are common in littoral salt marshes on temporally flooded humid soils. Although they often share the same habitats they have different ecological optima. *J. maritimus* is more salt tolerant than *J. acutus* (Boscaiu *et al.* 2011, 2013) and restricted to saline humid soils whilst *J. acutus* is found mostly on salt marsh borders or other areas with lower salinity. Since *J. acutus* also tolerates drought relatively well, it is to be found in soil with a sandy texture such as that occurring in small depressions among dunes or on gypsum soils (Boira 1995). *J. articulatus* is a more salt-sensitive species that occupies river banks and non-saline wetlands (Albrecht 1994).

This study compares oxidative stress defence mechanisms induced by drought or soil salinity, in *J. maritimus, J. acutus* and *J. articulatus*, with the aim of generating novel information on the general mechanisms of stress tolerance in this genus and perhaps more widely. Our working hypothesis is that the activation of antioxidant responses contributes significantly to the mechanisms of salt and drought tolerance in *Juncus*. For this to be correct, stress-induced levels of antioxidant compounds and activities of antioxidant enzymes should correlate with the relative stress tolerance, i.e. they should be higher in the halophytes *J. maritimus* and *J. acutus* than in the more sensitive *J. articulatus.*

We analysed: (i) stress-induced inhibition of vegetative growth (plant shoot fresh weight (FW), water content (WC)) and levels of photosynthetic pigments, (ii) accumulation of malondialdehyde (MDA) as a reliable oxidative stress marker (iii) total phenolic compounds (TPC) and flavonoid contents, as examples of non-enzymatic antioxidants and (iv) specific activities of four antioxidant enzymes: SOD, CAT, GR and APX. The results of these analyses were correlated with the relative stress tolerance of the investigated species, estimated from their distribution in nature and their stress-induced growth inhibition under controlled experimental conditions.

## Methods

### Plant material and experimental design

Seeds of *J. acutus*, *J. maritimus* and *J. articulatus* were collected in ‘La Albufera’ Natural Park (Province of Valencia, Spain). After sterilization with commercial bleach and repeated washes with distilled water, seeds were sown on a mixture of commercial peat:perlite:vermiculite (2:1:1), in 1 L pots placed in plastic trays (12 pots per tray), and watered twice per week with 1.5 L of half strength Hoagland nutrient solution (Hoagland and Arnon 1950) added to each tray. After 6 weeks, control, salt and drought treatments were initiated and carried out for 8 weeks. Plants subjected to salt stress were watered twice a week with the same volume of the nutrient solution containing NaCl at 100, 200 or 400 mM. Drought treatments were started at the same time by withholding irrigation. For each treatment (control, water stress and various NaCl concentrations), five individual plants of each species were used as biological replicates.

All experiments were conducted in a controlled environment chamber, under the following conditions: long-day photoperiod (16 h of light), at 23 °C during the day and 17 °C at night, a CO_2_ level of ∼300 ppm and 50–80 % relative humidity. Plant material (the aerial part of each plant) was harvested after 8 weeks.

### Soil analysis

Electrical conductivity (EC_1:5_) of the substrate in all pots was determined at the end of the experiment. Soil samples were air-dried, passed through a 2-mm sieve, mixed with deionized water in a proportion of 1–5, and the suspensions stirred for 1 h at 600 rpm, at room temperature. Electric conductivity was measured with a Crison Conductivity meter 522 and expressed in dS m^−1^.

### Growth measurements

The FW of the aerial part of each plant was measured and a fraction of the material was dried at 65 °C for 4 days to constant weight and weighed again for dry weight (DW). The WC of each sample was calculated as (FW – DW). To facilitate comparisons between the three *Juncus* species, which differ in size and show slightly different shoot WC under control conditions, FW and WC values were expressed as percentage of the mean FW or WC of corresponding control plants. Absolute FW and WC mean values of the non-treated controls are indicated in the legend of Table 2.

### Quantification of photosynthetic pigments

Total carotenoids (Caro), chlorophyll a (Chl a) and chlorophyll b (Chl b) were measured following Lichtenthaler and Wellburn (1983): 100 mg of fresh shoot material were ground in 30 mL of ice-cold 80 % acetone, mixed by vortexing and then centrifuged. Absorbance of the supernatant was measured at 663, 646 and 470 nm, and the concentration of each group of compounds was calculated according to the following equations:

$$\begin{array}{l} \text{Chl }a\;(\mu g\;{\text{mL}}^{-1})\;=\;12.21\;\left(A_{663}\right)\;-\;2.81\;\left(A_{646}\right),\\ \text{Chl }b\;(µg\;{\text{mL}}^{-1})\;=\;20.13\;\left(A_{646}\right)\;-\;5.03\;\left(A_{663}\right),\\ \text{Caro }(µg\;{\text{mL}}^{-1})\;=\;(1000A_{470}\;-\;3.27[\text{Chl achl }a]\;-\;104\left[\text{Chl }b\right])/227. \end{array}$$

Pigment concentrations were expressed in mg g^−1^ DW.

### MDA and non-enzymatic antioxidants

Dried plant material was extracted with 80 % methanol, in a rocker shaker, for 24–48 h. MDA in the extracts was determined using a previously described method (Hodges *et al.* 1999). Briefly, the samples were mixed with 0.5 % thiobarbituric acid (TBA) prepared in 20 % TCA (or with 20 % TCA without TBA for the controls), and then incubated at 95 °C for 20 min. After stopping the reaction on ice, the absorbance of the supernatants was measured at 532 nm. The non-specific absorbance at 600 and 440 nm was subtracted and MDA concentration determined using equations from Hodges *et al.* (1999).

TPC were quantified according to Blainski *et al.* (2013), by reaction with the Folin–Ciocalteu reagent. The methanol extracts were mixed with sodium bicarbonate and the reagent and absorbance was recorded at 765 nm using gallic acid (GA) as standard. The measured TPC concentrations were expressed as GA equivalents (mg eq. GA g^−1^ DW).

Total ‘antioxidant flavonoids’ (TF) were determined by a reaction with NaNO_2_ and AlCl_3_ at a basic pH, as described by Zhishen *et al.* (1999), with catechin used as standard; the product of the reaction being detected spectrophotometrically, at 510 nm. This protocol is often claimed to measure ‘total flavonoids’ in the sample, although this is not strictly true. The method is based on the nitration of aromatic rings containing a catechol group. Several groups of flavonoids—e.g. flavonols and flavanols—but also other phenolics, such as caffeic acid and derivatives react in this way. Nevertheless, phenolic compounds detected in the assay are all strong antioxidants and there is a good correlation between their levels and the total antioxidant activity of the samples (Zhishen *et al.* 1999). To simplify, in the text we refer to the AlCl_3_-reactive compounds simply as TF, and express their concentrations as equivalents of catechin (mg eq. C g^−1^ DW).

### Protein extraction and quantification

Crude protein extracts were prepared from fresh plant material as described by Gil *et al.* (2014). Samples were ground in the presence of liquid N_2_ and then mixed with extraction buffer [20 mM Hepes, pH 7.5, 50 mM KCl, 1 mM EDTA, 0.1 % (v/v) Triton X-100, 0.2 % (w/v) polyvinylpyrrolidone, 0.2 % (w/v) polyvinylpolypyrrolidone and 5 % (v/v) glycerol]. A 1/10 volume of ‘high salt buffer’ (225 mM Hepes, pH 7.5, 1.5 M KCl and 22.5 mM MgCl_2_) was added to each sample, and the homogenates were centrifuged for 20 min at 20 000 *g* and 4 °C. Supernatants were collected, concentrated in U-TubeTM concentrators (Novagen, Madison, USA), and centrifuged to remove precipitated material. The final samples (referred to as ‘protein extracts’) were divided into aliquots, flash-frozen in liquid N_2_ and stored at -75 °C until used for enzyme assays. Protein concentration in the extracts was determined by the method of Bradford (1976), using bovine serum albumin as a standard and the Bio-Rad commercial reagent.

### Antioxidant enzyme activity assays

SOD activity in the protein extracts was determined according to Beyer and Fridovich (1987) by following spectrophotometrically (at 560 nm) the inhibition of nitroblue tetrazolium (NBT) photoreduction, in reaction mixtures containing riboflavin as the source of superoxide radicals. One SOD unit was defined as the amount of enzyme causing 50 % inhibition of NBT photoreduction under the assay conditions.

CAT activity was determined as described by Aebi (1984), following the decrease in absorbance at 240 nm which accompanies the consumption of H_2_O_2_ added to protein extracts. One CAT unit was defined as the amount of enzyme that will decompose 1 µmol of H_2_O_2_ per minute at 25 °C.

APX activity was determined according to Nakano and Asada (1981) by measuring the decrease in absorbance at 290 nm as ascorbate becomes oxidized in the reaction. One APX unit was defined as the amount of enzyme required to consume 1 µmol of ascorbate per minute, at 25 °C.

GR activity was determined according to Connell and Mullet (1986), following the oxidation of NADPH [the cofactor in the GR-catalysed reduction of oxidized glutathione (GSSG)] by the decrease in absorbance at 340 nm. One GR unit was defined as the amount of enzyme that will oxidize 1 µmol of NADPH per minute at 25 °C.

Minor modifications to the original enzymatic assays are described in Gil *et al.* (2014).

### Data analysis

Data were analysed using Statgraphics Centurion XVI. Before the analysis of variance, the Shapiro–Wilk test was used to check for validity of normality assumption and Levene’s test for the homogeneity of variance. If ANOVA requirements were met, significant differences among treatments were tested by one-way ANOVA at the 95 % confidence level and *post hoc* comparisons were made using the Tukey HSD test. All means throughout the text are followed by their SD (*n* = 5). A two-way ANOVA was applied to test the effect of species, treatments (salt and drought, separately) and their interaction on the analysed characteristics.

In addition, all parameters measured in plants submitted to salt stress treatments were correlated by principal component analysis (PCA), independently for each of the three studied *Juncus* species. Each principal component was a linear combination of the original variables with coefficients equal to the eigenvectors of the correlation matrix. A graphic interpretation was obtained as a biplot on the 2D of the main principal components.

## Results

### Salt stress

***EC of substrates.*** EC_1:5_ was measured in samples of the pot substrates, once the 8-week salt and water deficiency treatments had been concluded. At the end of the experiment, a similar increase in EC_1:5_ was detected in parallel to the increase of NaCl concentrations in the soil of all three *Juncus* species. Conductivities reached between 13 and 14 dS m^−1^ in pots watered with nutrient solution containing 400 mM NaCl (Table 1) thus confirming the high correlation between EC_1:5_ and the concentration of the saline solution. As expected, water deficiency did not modify EC in the pots (data not shown).

**Table 1.** Electrical conductivity (EC_1:5_, dS m^−1^) of pot substrate samples after 8-week treatments with the indicated NaCl concentrations, for the three *juncus* species under study. The values shown are means with SD (*n* = 5). Different lowercase letters in each column indicate significant differences between salt treatments applied to a species, and different capital letters in each row indicate significant differences between species undergoing the same salt treatment, according to the Tukey test (*α* = 0.05). The results of one-way ANOVAs carried out for each species ([*P*-values, *F*-ratios, and ‘degrees of freedom’ (*df*)] are shown below the main table.

	EC_1:5_ (dS m^−1^)		
NaCl treatments (mM)	*J. maritimus*	*J. acutus*	*J. articulatus*
0	1.10 ± 0.20aA	1.00 ± 0.10aA	1.00 ± 0.20aA
100	5.30 ± 0.40bA	5.10 ± 0.40bA	4.80 ± 0.60bA
200	7.50 ± 0.40cA	7.70 ± 0.50cA	9.70 ± 0.80cB
400	13.40 ± 0.50dAB	14.30 ± 0.50dB	13.00 ± 0.40dA
**One-way ANOVA**	***J. maritimus***	***J. acutus***	***J. articulatus***
*P* value	0.0000	0.0000	0.0000
*F* ratio	956.02	1029.57	514.56

df between groups: 3.

df within groups: 16.

**Table 2.** Variation in (A) fresh weight (FW, %) and (B) relative water content (WC, %), in shoots of *J. maritimus*, *J. acutus*, and *J. articulatus* plants after 8 weeks of salt treatments with the indicated concentrations of NaCl. The mean FW (A) and WC (B) of control, non-treated plants (*J. maritimus*: 3.85 g, 78 %; *J. acutus*: 2.60 g, 81 %; *J. articulatus*: 21.69 g, 86 %, respectively) were considered as 100 % for each species. The values shown are means with SD (*n* = 5). For each species, different lowercase letters indicate significant differences between treatments according to the Tukey test (*α* = 0.05). The results of one-way ANOVAs carried out for each species [*P*-values, *F*-ratios, and ‘degrees of freedom’ (*df*)] are shown below the main table.

**(A)**	Fresh weight (%)		
NaCl treatments (mM)	*J. maritimus*	*J. acutus*	*J. articulatus*
0	100.00c	100.00b	100.00c
100	53.00 ± 10.00b	43.00 ± 4.00a	38.00 ± 8.00b
200	45.00 ± 15.00ab	34.00 ± 3.00a	10.00 ± 4.00a
400	29.00 ± 9.00a	33.00 ± 3.00a	9.00 ± 3.00a
**(B)**	**Water content (%)**		
**NaCl treatments (mM)**	***J. maritimus***	***J. acutus***	***J. articulatus***

0	100.00c	100.00c	100.00b
100	96.00 ± 4.00b	94.00 ± 4.00bc	92.00 ± 7.00b
200	95.00 ± 9.00b	90.00 ± 9.00b	82.00 ± 6.00ab
400	83.00 ± 2.00a	81.00 ± 4.00a	75.00 ± 8.00a

	One-way ANOVA	*J. maritimus*	*J. acutus*	*J. articulatus*
FW %	*P* value	0.0001	0.0000	0.0000
	*F* ratio	14.91	60.09	26.00
WC %	*P* value	0.0000	0.0000	0.0000
	*F* ratio	64.65	73.29	74.48


df between groups: 3.

df within groups: 16.

***Growth parameters***. The FW of aerial parts as a percentage of appropriate unstressed controls was reduced under salt stress in each species but with quantitative differences between the taxa. Reduction in biomass accumulation was more pronounced in salt-sensitive *J. articulatus* than in the two halophytes. Thus, in the presence of 100 mM NaCl, FW of the shoots of *J. articulatus* was 38 % of controls compared with 53 % and 43 % for *J. maritimus* and *J. acutus*, respectively. At the highest salt concentration (400 mM) relative fresh mass was reduced by >90 % in *J. articulatus*, compared with ∼70 % in *J. maritimus* and *J. acutus* (Table 2A). Therefore, the relative salt tolerance of *J. maritimus *>* J. acutus *>* J. articulatus* as defined by the degree of salt-induced inhibition of growth, correlates well with their distribution in nature. Similarly, *Juncus* taxa were quite resistant to salt-induced dehydration in terms of the relative reductions of WC, with water losses of 17 %, 19 % and 25 % for *J. maritimus*, *J. acutus* and *J. articulatus*, respectively, in the presence of 400 mM NaCl (Table 2B). These values correlate negatively with the salt tolerance of the species in the wild.

**Figure 1:**
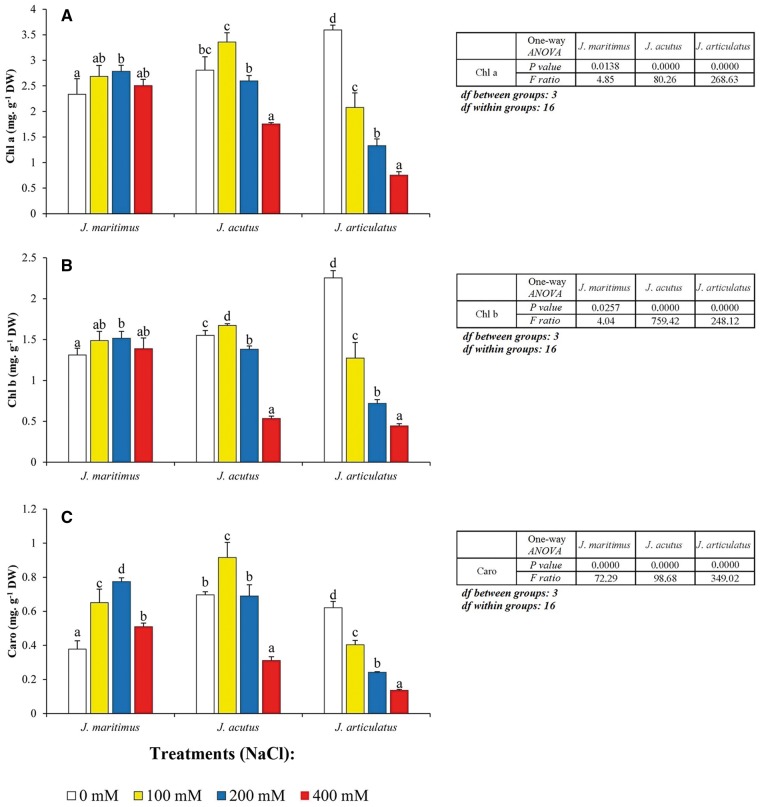
Photosynthetic pigments in three *Juncus* species after 8-week treatment with a range of NaCl concentrations. Leaf concentrations of (A) chlorophyll a, (B) chlorophyll b and (C) total carotenoids, are shown as means with SD (*n* = 5). For each species, different letters above the bars indicate significant differences between treatments according to the Tukey test (*α* = 0.05). The results of the corresponding one-way ANOVAs [*P values*, *F ratios*, *degrees of freedom (df)*] are shown beside each graph.

The general patterns of salt-induced changes in the shoot levels of photosynthetic pigments were also clearly different in the halophytes and salt-sensitive species (Fig. 1). In *J. articulatus*, a concentration-dependent reduction of Chl a (Fig. 1A), Chl b (Fig. 1B) and Caro (Fig. 1C) concentrations with increasing external NaCl concentrations, was measured. In contrast, chlorophyll levels remained almost constant in the most salt-tolerant species, *J. maritimus* whilst *J. acutus* showed intermediate behaviour with a significant reduction of chlorophylls taking place only under the two highest salinity levels (Fig. 1A and B). Changes in Caro concentrations were broadly similar to those seen for chlorophyll but with the concentrations boosted by 100* *mM and/or 200* *mM in the two halophytes and a fall in 400* *mM NaCl (Fig. 1C). As shown in Table 3, a two-way ANOVA revealed significant differences, according to ‘treatment’, ‘species’ and significant interactions between the two independent variables, for all growth parameters (FW, WC) and photosynthetic pigments (Chl a, Chl b, Caro).

**Table 3.** Salt treatments of the selected *juncus* species. Results of two-way ANOVAs (*P* values, *F* ratios) for the independent variables ‘species’ and ‘treatment’, and the ‘species × treatment’ interactions. The measurements included were: fresh weight (FW), water content percentage (WC %), chlorophyll a (chl a), chlorophyll b (chl b), total carotenoids (caro), malondialdehyde (MDA), total phenolic compounds (TPC), total flavonoids (TF), and the specific activities of superoxide dismutase (SOD), catalase (CAT), ascorbate peroxidase (APX), and glutathione reductase (GR).

*Measured parameter*	Species	Treatment	Interaction
FW	*P*: 0.0000	*P*: 0.0000	*P*: 0.0000
	*F*: 55.31	*F*: 37.12	*F*: 20.16
WC %	*P*: 0.0005	*P*: 0.0000	*P*: 0.0000
	*F*: 9.08	*F*: 197.86	*F*: 9.27
Chl a	*P*: 0.0000	*P*: 0.0000	*P*: 0.0000
	*F*: 91.39	*F*: 140.78	*F*: 83.04
Chl b	*P*: 0.0000	*P*: 0.0000	*P*: 0.0000
	*F*: 37.84	*F*: 275.49	*F*: 129.78
Caro	*P*: 0.0000	*P*: 0.0000	*P*: 0.0000
	*F*: 250.57	*F*: 165.03	*F*: 92.89
MDA	*P*: 0.0000	*P*: 0.0000	*P*: 0.0000
	*F*: 219.41	*F*: 255.43	*F*: 161.47
TPC	*P*: 0.0000	*P*: 0.0000	*P*: 0.0000
	*F*: 18.06	*F*: 408.49	*F*: 129.34
TF	*P*: 0.0000	*P*: 0.0000	*P*: 0.0000
	*F*: 57.31	*F*: 430.42	*F*: 35.03
SOD	*P*: 0.0000	*P*: 0.0000	*P*: 0.0000
	*F*: 1465.98	*F*: 61.38	*F*: 84.12
CAT	*P*: 0.0000	*P*: 0.0000	*P*: 0.0000
	*F*: 196.76	*F*: 136.93	*F*: 27.52
APX	*P*: 0.0000	*P*: 0.0000	*P*: 0.0000
	*F*: 219.85	*F*: 51.19	*F*: 120.82
GR	*P*: 0.0000	*P*: 0.0000	*P*: 0.0000
	*F*: 155.20	*F*: 107.53	*F*: 49.08

#### MDA and non-enzymatic antioxidants

**Figure 2:**
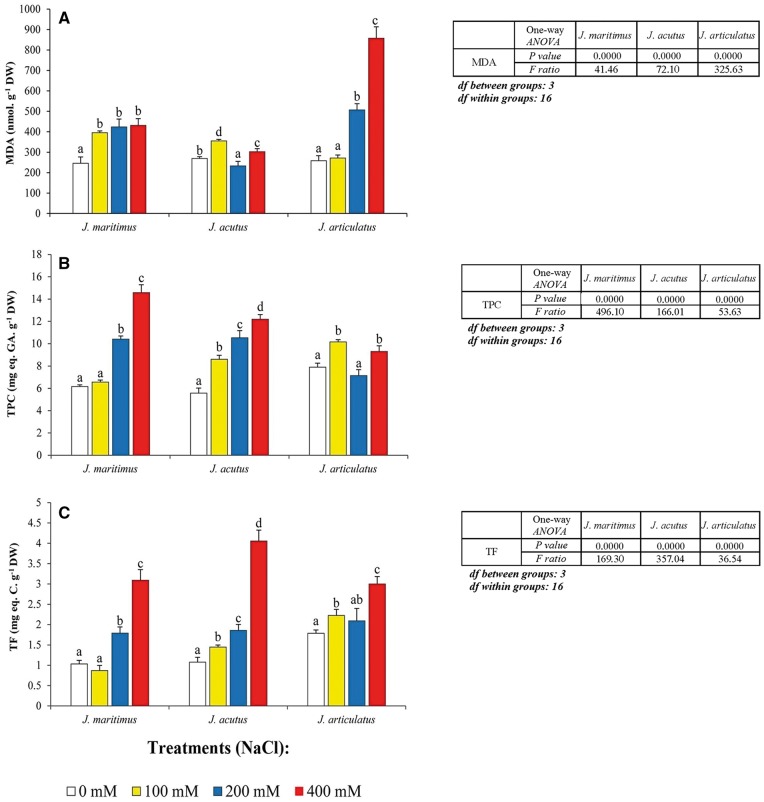
Oxidative stress marker and non-enzymatic antioxidants in three *Juncus* species, after 8-week treatment with a range of NaCl concentrations. Leaf concentrations of (A) malondialdehyde (MDA), (B) total phenolic compounds (TPC) and (C) total flavonoids (TF), are shown as means with SD (*n* = 5). For each species, different letters above the bars indicate significant differences between treatments, according to the Tukey test (*α* = 0.05). The results of the corresponding one-way ANOVAs [*P values, F ratios, degrees of freedom (df)*] are shown beside each graph.

MDA concentrations differed little in the three *Juncus* taxa under non-stress conditions and increased only slightly in halophytes *J. maritimus* and *J. acutus* under salt stress. However, in salt-sensitive *J. articulatus*, 200* *mM and 400* *mM NaCl raised MDA concentrations, with a 3-fold increase occurring in the latter (Fig. 2A). TPC and TF levels were similar in control *J. maritimus* and *J. acutus* and increased significantly in parallel with increasing salinity (Fig. 2B and C). In *J. articulatus*, TPC levels did not show a clear correlation with NaCl concentrations (Fig. 2B), whilst salt-induced increases of TF were much smaller than in *J. maritimus* or *J. acutus* at 400 mM (Fig. 2C). According to the results of two-way ANOVA, the levels of all analysed compounds (MDA, TPC and TF) were significantly different in respect of species, treatments, and their interactions (Table 3).

#### Antioxidant enzyme activities

**Figure 3:**
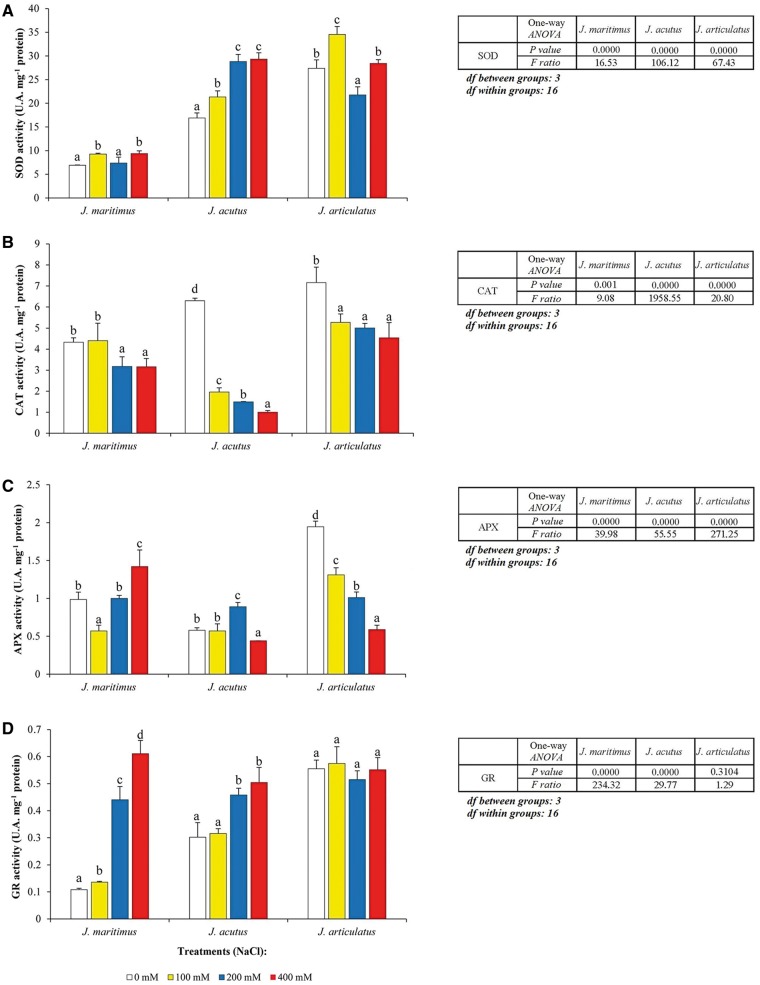
Activity of antioxidant enzymes in three *Juncus* species, after 8-week treatments with a range of NaCl concentrations. The graphs show specific activities of (A) superoxide dismutase (SOD), (B) catalase (CAT), (C) ascorbate preoxidase (APX) and (D) glutathione reductase (GR), as mean values with SD (*n* = 5). For each species, different letters above the bars indicate significant differences between treatments, according to the Tukey test (*α* = 0.05). The results of the corresponding one-way ANOVAs [*P values, F ratios, degrees of freedom (df)*] are shown beside each graph.

The specific activities of several antioxidant enzymatic systems showed different variation patterns in response to the salt treatments, depending on species and enzyme (Fig. 3). SOD activity tended to rise under salt in each species although the trend was clearest in *J. acutus* where activity was increased 75 % by 400 mM NaCl (Fig. 3A). Activities of CAT were depressed by salt most strongly in *J. acutus* (up to 85 % less) and *J. articulatus* (up to 34 % inhibition) with a smaller reduction seen in the most salt-tolerant *J. maritimus* (max. inhibition of 25 %) (Fig. 3B). The inherently high APX activities of salt-sensitive *J. articulatus* were depressed by up to 71 % as salt concentrations increased. No clear trends were found in *J. acutus* and *J. maritimus* except at 400* *mM salt when APX in *J. maritimus* rose by a third (Fig. 3C). The strongest response to salt was seen in GR activity of *J. maritimus* which was stimulated almost 6-fold by 400* *mM NaCl. Smaller increases were seen in the less salt tolerant *J. acutus* but no changes in activity in salt-sensitive *J. articulatus* (Fig. 3D). Two-way ANOVA revealed that all enzymatic activities (SOD, CAT, APX, GR) differed significantly between species and treatments (*P* < 0.05), and that the interaction of the two independent variables was also statistically significant (Table 3).

#### PCAs

**Figure 4:**
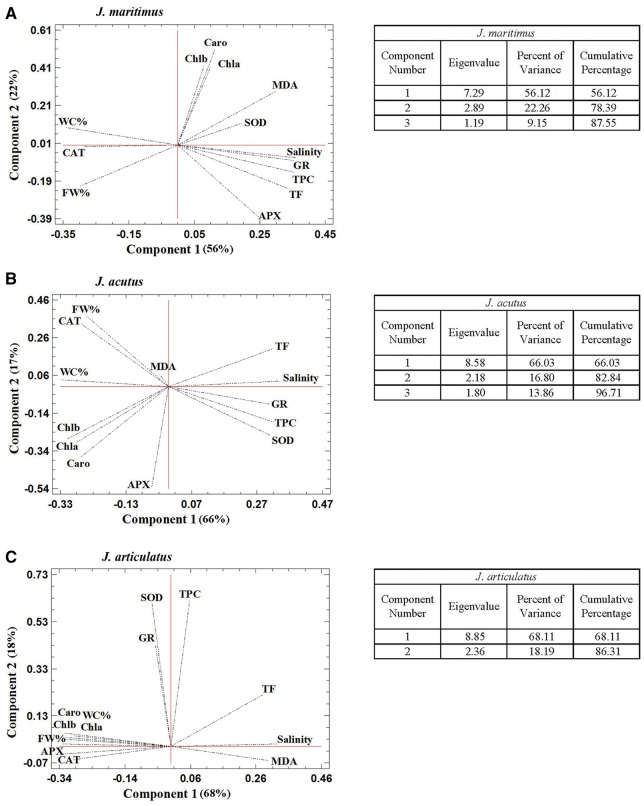
Principal component analysis (PCA). Site score plots of the studied variables in the salt stress treatments, for the three *Juncus* species, *J. maritimus* (A), *J. acutus* (B) and *J. articulatus* (C). PCAs included, as analysed variables: substrate EC_1:5_ (Salinity), water content (WC %), fresh weight (FW %), chlorophyll a (Chl a), chlorophyll b (Chl b), total carotenoids (Caro), malondialdehyde (MDA), total phenolic compounds (TPC), total flavonoids (TF), and specific activities of superoxide dismutase (SOD), catalase (CAT), ascorbate peroxidase (APX) and glutathione reductase (GR).

Principal component analyses, including all parameters measured under salt stress, were performed independently for *J. maritimus* (Fig. 4A), *J. acutus* (Fig. 4B), and *J. articulatus* (Fig. 4C). Three components with an Eigenvalue >1 explained 88 % of the data variability in *J. maritimus* and 97 % in *J. acutus*. In *J. articulatus*, only two components had an Eigenvalue >1, explaining 86 % of the variability. In all PCAs, the first component (*X*-axis) was determined by the ‘salinity’ variable (established by EC_1:5_ measurements in the pot substrates, Table 1). Correlation of the analysed parameters with substrate salinity varied, depending on the relative salt tolerance of the species. In the three PCAs, growth parameters (FW, WC) were found to be negatively correlated with salinity, as salt stress inhibited growth in all cases. However, the corresponding loading vectors presented smaller angles with the *X*-axis in *J. articulatus* than in the two salt-tolerant taxa, indicating a stronger negative correlation with EC. Similarly, the negative correlation between salinity and variation of photosynthetic pigments levels was much stronger in *J. articulatus* than in *J. acutus*, whilst no significant correlation was detected in *J. maritimus* (Fig. 4).

There were other clear differences between the three species: MDA, considered a reliable indicator of oxidative stress, was strongly positively correlated with salinity only in *J. articulatus*. This species also showed a strong negative correlation between the substrate EC and APX and CAT activities. Salt-sensitive *J. articulatus* also gave a relatively weak positive correlation with antioxidant TF contents, but no significant correlation with TPC levels or SOD and GR activities. On the contrary, in the halophytes *J. maritimus* and *J. acutus*, positive correlations between salinity and TPC concentrations or SOD and GR activities were detected (Fig. 4).

### Drought stress

***Growth parameters***. Drought stress inhibited growth of all three *Juncus* species. As under salt stress, *J. articulatus* was the most affected by water shortage. Eight weeks after last watering, mean shoot FW was reduced to ca. 3 % of the corresponding well-watered control. Strong relative decreases in FW were also observed in *J. maritimus* (down to 12 % of the value measured in the non-stressed plants) and in *J. acutus* (17 % of the control), which appears to be the most drought-tolerant species (Table 4A). However, a fraction of this FW reduction was not due strictly to growth inhibition, but to loss of water (Table 4B); drought-induced dehydration of the aerial part of the plants being stronger than that observed in salt-stressed plants, even in the presence of 400 mM NaCl (compare Tables 4B and 2B).

**Table 4.** Variations in (A) fresh weight (FW, %) and (B) relative water content (WC, %), in shoots of *J. maritimus*, *J. acutus*, and *J. articulatus* plants after 8-week water-deficiency stress (WS) treatment. The mean FW (A) and WC (B) of control plants, which were considered as 100 % for each species, are indicated in the legend of Table 2. The values shown are means with SD (*n* = 5). For each species, different lowercase letters indicate significant differences between treatments according to the Tukey test (*α* = 0.05). The results of one-way ANOVAs carried out for each species [*P*-values, *F*-ratios, and ‘degrees of freedom’ (*df*)] are shown below the main table.

(A)	Fresh weight (%)		
Treatments	*J. maritimus*	*J. acutus*	*J. articulatus*
Control	100.00b	100.00b	100.00b
WS	11.90 ± 1.60a	16.90 ± 3.80a	3.30 ± 0.70a
**(B)**	**Water content (%)**		
**Treatments**	***J. maritimus***	***J. acutus***	***J. articulatus***

Control	100.00b	100.00b	100.00b
WS	31.00 ± 4.80a	11.30 ± 0.60a	18.50 ± 2.50a

	One-way ANOVA	*J. maritimus*	*J. acutus*	*J. articulatus*
FW %	*P* value	0.0000	0.0000	0.0000
	*F* ratio	64.65	73.29	74.48
WC %	*P* value	0.0000	0.0000	0.0000
	*F* ratio	102.52	936.52	186.73


df between groups: 1.

df within groups: 8.

Photosynthetic pigments (Chl a, Chl b and Caro) decreased in droughted plants of each species, relative to controls (Table 5). This reduction in chlorophyll was most pronounced in *J. articulatus* (80 % of the corresponding controls), whilst in *J. maritimus* the reduction was ∼50 % and 40 % in *J. acutus* (Table 5A and B). Decreases in Caro were also large, dropping by ∼83 % in *J. articulatus*, 71 % in *J. acutus* and by 50 % in *J. maritimus* (Table 5C). Two-way ANOVA revealed significant differences, at the 95 % confidence level, attributable to ‘treatment’, ‘species’ and their interactions, for all growth parameters and photosynthetic pigments analysed, except for the differences of WC between species (Table 6).

**Table 5.** Photosynthetic pigments in the shoots of the three *juncus* species under study, after 8-week water-deficiency stress (WS) treatment. Variations in (A) chlorophyll a, (B) chlorophyll b, and (C) total carotenoid contents. The values shown are means with SD (*n* = 5). For each species, different lowercase letters indicate significant differences between treatments according to the Tukey test (*α* = 0.05). The results of one-way ANOVAs carried out for each species [*P*-values, *F*-ratios, and ‘degrees of freedom’ (*df*)] are shown below the main table.

(A)	Chlorophyll a (mg g^−1^ DW)		
Treatments	*J. maritimus*	*J. acutus*	*J. articulatus*
Control	2.30 ± 0.30b	2.80 ± 0.20b	3.60 ± 0.10b
WS	1.20 ± 0.10a	1.70 ± 0.10a	0.80 ± 0.10a
**(B)**	**Chlorophyll b (mg g^−1^ DW)**		
**Treatments**	***J. maritimus***	***J. acutus***	***J. articulatus***

Control	1.30 ± 0.10b	1.60 ± 0.10b	2.30 ± 0.10b
WS	0.60 ± 0.10a	0.90 ± 0.10a	0.50 ± 0.10a

**(C)**	**Total carotenoids (mg g^−1^ DW)**		
**Treatments**	***J. maritimus***	***J. acutus***	***J. articulatus***

Control	0.40 ± 0.10b	0.70 ± 0.00b	0.60 ± 0.00b
WS	0.20 ± 0.00a	0.20 ± 0.00a	0.10 ± 0.00a

	One-way ANOVA	*J. maritimus*	*J. acutus*	*J. articulatus*
**(A)** Chl a	*P value*	0.0000	0.0000	0.0000
	*F ratio*	70.67	98.50	3094.63

**(B)** Chl b	*P value*	0.0000	0.0000	0.0000
	*F ratio*	217.57	275.21	1415.38

**(C)** Caro	*P value*	0.0000	0.0000	0.0000
	*F ratio*	37.76	3240.00	482.50


df between groups: 1.

df within groups: 8.

**Table 6.** Drought treatments of the selected *juncus* species. Results of two-way ANOVAs (*P* values, *F* ratios) for the independent variables ‘species’ and ‘treatment’, and their interactions. Abbreviations of the measured parameters (dependent variables) as in the legend of Table 3.

Measured parameter	Species	Treatment	Interaction
FW	*P*: 0.0000	*P*: 0.0000	*P*: 0.0000
	*F*: 28.04	*F*: 56.03	*F*: 26.45
WC (%)	*P*: 0.1389	*P*: 0.0000	*P*: 0.0131
	*F*: 2.15	*F*: 640.43	*F*: 5.22
Chl a	*P*: 0.0000	*P*: 0.0000	*P*: 0.0000
	*F*: 22.16	*F*: 768.49	*F*: 84.25
Chl b	*P*: 0.0000	*P*: 0.0000	*P*: 0.0000
	*F*: 82.63	*F*: 1617.49	*F*: 187.83
Caro	*P*: 0.0000	*P*: 0.0000	*P*: 0.0000
	*F*: 32.55	*F*: 1223.55	*F*: 126.06
MDA	*P*: 0.0000	*P*: 0.0000	*P*: 0.0000
	*F*: 67.04	*F*: 646.89	*F*: 50.54
TPC	*P*: 0.0008	*P*: 0.0000	*P*: 0.0000
	*F*: 9.76	*F*: 208.55	*F*: 52.50
TF	*P*: 0.0000	*P*: 0.0000	*P*: 0.239
	*F*: 49.84	*F*: 857.91	*F*: 1.52
SOD	*P*: 0.0000	*P*: 0.0000	*P*: 0.0000
	*F*: 893.18	*F*: 455.35	*F*: 140.85
CAT	*P*: 0.0000	*P*: 0.0000	*P*: 0.0000
	*F*: 136.47	*F*: 712.44	*F*: 28.43
APX	*P*: 0.0000	*P*: 0.0000	*P*: 0.0000
	*F*: 244.52	*F*: 370.71	*F*: 356.18
GR	*P*: 0.0000	*P*: 0.0000	*P*: 0.4755
	*F*: 147.63	*F*: 345.98	*F*: 0.77

**Figure 5:**
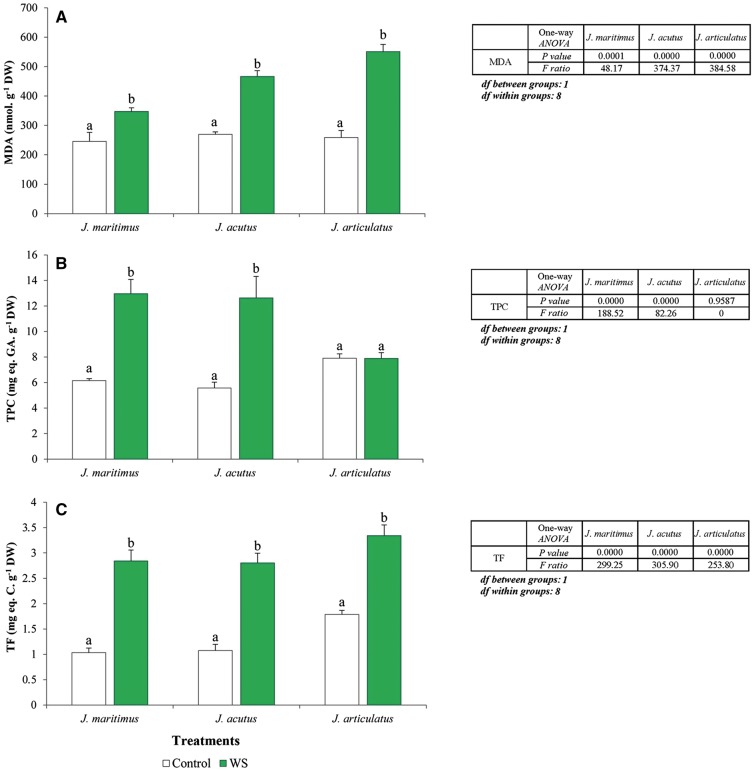
Oxidative stress and non-enzymatic antioxidants in shoots of three *Juncus* species, after an 8-week water-deficiency treatment. Shoot system concentrations of (A) malondialdehyde (MDA), (B) total phenolic compounds (TPC) and (C) total flavonoids (TF) are shown as means with SD (*n* = 5). For each species, different letters above the bars indicate significant differences in between treatments according to the Tukey test (*α* = 0.05). The results of the corresponding one-way ANOVAs [*P values, F ratios, degrees of freedom (df)*] are shown beside each graph.

***MDA and non-enzymatic antioxidants***. Water deficiency generated oxidative stress as a secondary effect in each of the species, as revealed by statistically significant increases in MDA (Fig. 5A). Although MDA concentrations were similar in the non-treated controls of the three species, increase from water-deficient plants was greater in less salt-tolerant *J. articulatus* (> 2-fold), than in the halophytes *J. acutus* and *J. maritimus* (ca. 1.7- and 1.4-fold, respectively). Drought-induced changes in TPC were qualitatively like those observed in response to salt stress with TPC values more than doubling in stressed plants of *J. maritimus* and *J. acutus*, whilst remaining little changed in *J. articulatus* (Fig. 5B). TF concentrations increased in all three species after drought treatment and the effect was again more pronounced in the halophytes (2.6–2.8-fold) than in *J. articulatus* (1.9-fold) (Fig. 5C). Two-way ANOVA indicated significant differences for all compounds analysed (MDA, TPC and TF), regarding species, treatment and their interactions. An exception was for the interaction between the two independent variables in the case of TF, which was non-significant (Table 6).

**Figure 6:**
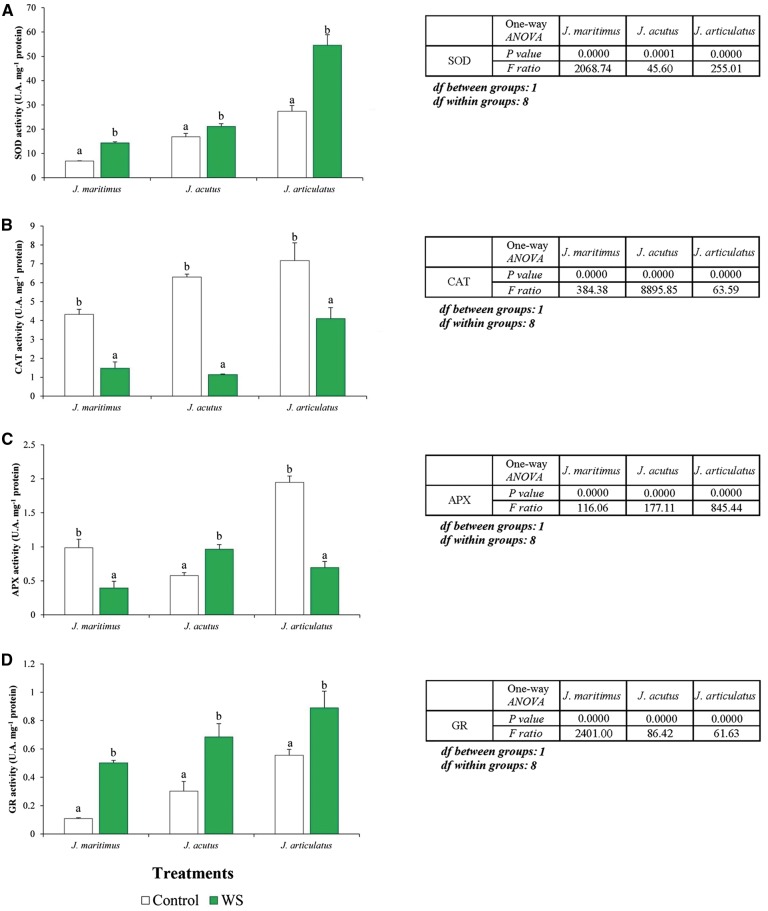
Activity of antioxidant enzymes in shoots of the three *Juncus* species after an 8-week water-deficiency treatment. The graphs show specific activities of (A) superoxide dismutase (SOD), (B) catalase (CAT), (C) ascorbate peroxidase (APX) and (D) glutathione reductase (GR), as mean values with SD (*n* = 5). For each species, different letters above the bars indicate significant differences in between treatments according to the Tukey test (*α* = 0.05). The results of the corresponding one-way ANOVAs [*P values, F ratios, degrees of freedom (df*)] are shown beside each graph.

***Antioxidant enzyme activities***. Antioxidant enzyme activities were altered by water deficiency in each species (Fig. 6), although patterns were quantitatively and qualitatively different from those observed under salt stress (Fig. 3). Notably, CAT activity was lowered by either stress treatment in each species (Figs 3B and 6B); the strongest drought-induced reduction in CAT activity being in *J. acutus*, followed by *J. maritimus* and *J. articulatus* (Fig. 6B). SOD and GR were activated in all three species by drought but there was no clear correlation between the relative stress tolerance of the different taxa and the activity increases over controls. Thus, SOD activity was almost doubled in *J. maritimus* and *J. articulatus*, whilst in *J. acutus* the increase was only ca. 1.2-fold (Fig. 6A). Maximum GR activation was detected in *J. maritimus* (almost 5-fold), followed by *J. acutus* (2.2-fold) and *J. articulatus* (1.6-fold) (Fig. 6D) whilst APX activity in *J. acutus* was raised 1.7-fold compared with controls but reduced to 35–40 % of control readings in *J. articulatus* and *J. maritimus* (Fig. 6C). For all enzymatic activities assayed here (SOD, CAT, APX, GR), significant differences regarding the independent variables ‘species’ and ‘treatment’ were revealed by two-way ANOVA; the interactions between the two factors were also significant for SOD, CAT and APX, but not for GR (Table 6).

## Discussion

Many publications report the activation of antioxidant systems in response to environmental conditions such as drought or soil salinity that generate oxidative stress. However, articles comparing antioxidant responses in genetically related wild species are rare, especially in monocots adapted to contrasting stressful habitats. The three *Juncus* taxa used in the present work help make good this deficiency in the literature, due to their differing tolerance, not only to salinity but also to drought and to their occurrence in neighbouring areas of the same geographic territory. In addition, salt tolerant *Juncus* have been little studied ecophysiologically and biochemically despite their importance as structural species in salt marshes and saline grasslands.

Water deficit in the soil due to lack of rain or irrigation (drought stress), or high concentration of salts in the soil solution (salt stress) both lower plant water potential, leading to cellular dehydration. High soil salinity has several additional deleterious effects for plants due to direct toxicity, mostly by Na^+^ ions. It has been considered that the initial response to salt stress is identical to that of drought, and is triggered by its osmotic component, whilst salinity-specific responses due to ion toxicity develop only over time with longer exposure to stress (Munns 2002). This overlap explains how the physiological effects of water deficiency and salinity can appear the same, by initiating a cascade of deleterious effects that hinder vegetative growth (Cramer *et al.* 1994; Hummel *et al.* 2010) mediated by ROS (Apel and Hirt 2004), nutritive imbalance (Grattan and Grieve 1994), desiccation and eventually plant death.

Growth inhibition is one of the first and most general responses to stress, as plants redirect their resources from primary metabolism and biomass accumulation to the activation of stress responses (Zhu 2001; Munns and Tester 2008). Reduction of growth under stressful conditions is more pronounced in stress sensitive than in stress tolerant plants (Demiral and Türkan 2005; Sekmen *et al.* 2007). As expected, halophytic *J. acutus* and *J. maritimus* proved to be more stress tolerant than *J. articulatus*—a species never found in naturally saline areas. Their greater tolerance was revealed in terms of a smaller reduction in FW and DW, and a relatively higher resistance to salt-induced dehydration.

Salt and drought stress also trigger the degradation of photosynthetic pigments, reducing the photosynthetic yield and, in the long term, contributing to stress lethality by lack of nutrients and energy. Monitoring the concentrations of photosynthetic pigments in stressed plants can be used as a biomarker of stress (Schiop *et al.* 2015). Here again, it seems logical to assume that any reduction should be more pronounced in less tolerant species (Li *et al.* 2006; Lee *et al.* 2007). Accordingly, we have observed a stress-dependent decrease in the levels of chlorophylls and carotenoids, in the three *Juncus* species, with the strongest changes detected, in general, in the more sensitive *J. articulatus*. These results are in agreement with numerous previous reports of decreased levels of photosynthetic pigments at high salinities in different plant taxa (e.g. Parida *et al.* 2004; Sai Kachout *et al.* 2013).

Previously, we investigated different stress responses, based on the control of ion transport and the accumulation of specific osmolytes in the same *Juncus* taxa and found that the higher tolerance of the halophytes is based, at least partly, on their greater ability to block transport of toxic ions to aerial parts and on the accumulation of much higher levels of proline (Al Hassan *et al.* 2016). The present work extends these studies, focusing on secondary oxidative stresses generated in the plants under saline and drought conditions.

Plants have developed a series of detoxification systems to counteract oxidative stress, by activating non-enzymatic and enzymatic mechanisms to reduce the levels of highly toxic ROS (Larkindale and Huang 2004). Our present results show that the most stress-sensitive species (*J. articulatus*) is more affected by water shortage- and salt-induced oxidative stress than the halophytes, *J. maritimus* and *J. acutus*. This was revealed by a stronger accumulation of MDA, a reliable marker of oxidative stress (Del Rio *et al.* 1996) presumably arising from a less efficient activation of antioxidant systems.

Phenolic compounds and especially certain flavonoids have numerous functions including adaptation to abiotic stresses (Farah and Donangelo 2006). Since many flavonoids and other phenolics are strong antioxidants, their accumulation can reduce oxidative damage (Hussain *et al.* 2013). Antioxidant flavonoid levels increased in all three *Juncus* taxa, in response to both, salt and drought, but the increase was stronger in the halophytes. These differences were even clearer when referring to stress-dependent accumulation of TPC, which also increased in salt-tolerant *J. maritimus* and *J. acutus*, whilst no significant changes were observed in salt-sensitive *J. articulatus*. These data point to a more efficient activation of non-enzymatic antioxidant systems in the more tolerant taxa. It should also be mentioned that the results reported here do not seem to agree with previous field studies carried out on *J. maritimus* and *J. acutus* growing in a littoral salt marsh near Valencia (east Spain). These showed much weaker, non-significant seasonal changes in TFC and TF content in the plants even though soil salinity and drought conditions varied widely through the year (Gil *et al.* 2014; Bautista *et al.* 2016). Strictly, it is unwise to compare experimental data obtained in the field and those derived from studies performed in controlled laboratory or greenhouse set-ups. The reasons include different developmental stage of the plants, the physical containment of the root systems in the pots as opposed to open growth in natural soil, etc. (see Boscaiu *et al.* 2013, for a more extensive discussion on this topic). Nevertheless, in the present case the most likely explanation of the disparity between the field and greenhouse results is that *J. acutus* and *J. maritimus* plants experienced much higher levels of stress in the artificial greenhouse treatments (eight consecutive weeks of water shortage and salt concentrations up to 400 mM NaCl) than they would usually experience in their natural habitats.

Antioxidant enzymes, such as SOD, CAT, APX or GR, among others, also constitute essential components in the machinery of defence against oxidative stress in plants (Gill and Tuteja 2010). There are relatively few published reports describing comparative studies on plant responses to abiotic stress based on the activation of antioxidant enzymes. Most of those studies have been carried out either on taxa belonging to different genera (Ellouzi *et al.* 2011; Srivastava *et al.* 2015) or, when dealing with congener species, mostly on dicots (Mittova *et al.* 2000, 2003; Sekmen *et al.* 2007). Data on monocotyledonous plants, derived from this kind of experimental approach, are much scarcer (e.g. Seckin *et al.* 2010).

SOD is probably the most effective enzymatic antioxidant, ubiquitous in all aerobic organisms prone to ROS-mediated oxidative stress; it is considered that the enzyme acts ‘in the first line of defence against oxidative stress in plants’ (Alscher *et al.* 2002; Larkindale and Huang 2004). SODs remove $O_{2}^{\text{–}}$ by catalysing its dismutation into less-toxic H_2_O_2_ and O_2_ (Gill and Tuteja 2010). SOD activity has been reported to increase under water deficiency (Sharma and Dubey 2005; Zlatev *et al.* 2006; Wang *et al.* 2008; Wang and Li 2008) and salt stress (Harinasut *et al.* 2003; Kukreja *et al.* 2005; Gapinska 2008) in a wide range of plant species, including stress tolerant and sensitive taxa. Positive and negative correlations, or no correlation at all, between SOD activity and salinity have been reported in glycophytes, whereas all available data in halophytes indicate an increase in SOD specific activity with increasing salt concentrations (Bose *et al.* 2014). CAT eliminates H_2_O_2_ by its dismutation into H_2_O and O_2_. Under stressful conditions, CAT activity has been reported to be up-regulated in a number of plant species (Eyidogan and Oz 2005; Yang *et al.* 2008), but down-regulated in others (e.g. Pan *et al.* 2006). APX has a higher affinity for H_2_O_2_ than CAT, and is thought to play an essential role in ROS scavenging during stress. APX was found to be activated under salt and drought stress in several species (Srivastava *et al.* 2005; Zlatev *et al.* 2006). Some halophytes have been shown to have higher APX activity than glycophytic related species (Mittova *et al.* 2000, 2003; Shalata *et al.* 2001; Sekmen *et al.* 2007). GR also helps to control redox status by maintaining the reduced form of glutathione (by reduction of GSSG) using NADPH as cofactor. Higher GR activity was reported in salt-stressed plants of the halophyte *Plantago maritima* compared with the related glycophyte *P. media* (Sekmen *et al.* 2007).

In the present study, it has not been possible to establish a general pattern of variation in the specific activities of antioxidant enzymes in response to salt and drought by the three *Juncus* species. The only common features seen were a reduction of CAT activities in the three taxa under saline- and water-deficient treatments and higher specific activities for the four enzymes tested in salt-sensitive *J. aritculatus*. The lower levels of CAT activity detected in salt-stressed and droughted plants, as compared with the controls, may reflect a highly dynamic antioxidant system in which levels of the active enzyme vary depending not only on the species but also on the intensity and/or duration of stress. For example, CAT may be inactivated under long-term stress, counteracting the initial, stress-induced activation of the expression of the corresponding gene. In this specific study, sampling of plant material was carried out after a long exposure to stress, but samplings at shorter times may have revealed an initial rise in CAT activity, as shown in other experiments (Kukreja *et al.* 2005). Yet, some general trends are discernible in terms of the relative changes in the activities of the different enzymes. For example, in salt-sensitive *J. articulatus*, salt stress did not activate any of the antioxidant enzymes. This agrees with the higher degree of oxidative stress in this species, revealed by its greater salt-induced MDA levels. Overall, SOD and GR activities were not changed significantly after 8 weeks of stress, whilst CAT and APX decreased in the presence of salt. This was confirmed by the corresponding PCA, which showed a strong negative correlation between salinity and CAT and APX, and no correlation with SOD and GR. Contrary to salt stress, water deficiency did induce a slight (< 2-fold) increase in SOD and GR activities in *J. articulatus*, indicating that the responses of this species to drought and salinity are similar, but not identical.

In *J. maritimus*, a strong increase in GR activity, and a weaker increase in SOD activity were detected in salt- and drought-treated plants. In *J. acutus*, the activation of SOD and GR was also observed in response to both stresses, although with some quantitative differences. Therefore, it seems that these two enzymes are involved in the mechanisms of stress tolerance in the *Juncus* halophytes, helping to maintain low levels of oxidative stress in the presence of salt and under drought conditions.

Notably, the patterns of changes in APX activity differed under salt and drought stress, and were opposite for the two halophytes. Thus, in *J. maritimus*, APX activity increased in the presence of salt, but decreased in water-deficient plants, whilst in *J. acutus* it was reduced in salt-stressed plants and increased under drought. This particular behaviour could be related to the specific ecological optima of the two halophytes and the characteristics of their preferred natural habitats. Thus, the activation of APX as a specific response to salinity in *J. maritimus* may contribute to the higher salt tolerance of this taxon, as compared with *J. acutus*. On the other hand, the latter species is better adapted to more arid soils, such as sand dunes, which could be partly dependent on drought-induced activation of APX.

## Conclusions

The halophytes *J. acutus* and *J. maritimus* are more tolerant to salt and drought than *J. articulatus*, as shown by their weaker inhibition of growth and smaller reduction of photosynthetic pigments contents under salt or drought stress. They are also less affected by oxidative stress under both conditions, as indicated by their lower stress-induced MDA levels. This lower degree of oxidative stress is explained by the more efficient activation of antioxidant mechanisms in *J. maritimus* and *J. acutus* than in *J. articulatus*. Accumulation of TPC and flavonoids was higher in stressed plants of the halophytic taxa, which also showed increases in SOD and GR activities. Although some responses were similar under salt and water deficient conditions, others were specifically associated to drought or salinity. In the most salt-sensitive *J. articulatus*, antioxidant enzymes (SOD and GR) are activated only in response to drought, but not to salinity. The stress-specific APX activation patterns in the halophytes *J. maritimus* and *J. acutus* may be related to the ecological optima of these species, as the former is more tolerant to salinity and the latter is better adapted to arid soils.

## Sources of Funding

This work was financed by internal funds for research support of the Polytechnic University of Valencia to M.P.D.-T., M.B. and O.V.

## Contributions by the Authors

M.P.D.-T. and M.B. supervised the greenhouse work. M.A.H. and J.C. performed the experiments. M.A.H., M.P.D.-T. and M.B. carried out the statistical analyses and preparation of figures. O.V. wrote the manuscript, with contributions from M.A.H. and M.B. O.V. and M.B. were responsible for the general design, coordination and supervision of the project.

## Conflicts of Interest Statement

None declared.
